# Diabetes Text-Message Self-Management Support Program (SMS4BG): A Pilot Study

**DOI:** 10.2196/mhealth.3988

**Published:** 2015-03-25

**Authors:** Rosie Dobson, Karen Carter, Richard Cutfield, Ashley Hulme, Richard Hulme, Catherine McNamara, Ralph Maddison, Rinki Murphy, Matthew Shepherd, Johan Strydom, Robyn Whittaker

**Affiliations:** ^1^National Institute for Health InnovationSchool of Population Health, Faculty of Medical and Health SciencesUniversity of AucklandAucklandNew Zealand; ^2^Waitemata District Health BoardAucklandNew Zealand; ^3^East Tamaki HealthcareAucklandNew Zealand; ^4^School of MedicineFaculty of Medical and Health SciencesUniversity of AucklandAucklandNew Zealand; ^5^School of Counselling, Human Services and Social WorkFaculty of EducationUniversity of AucklandAucklandNew Zealand

**Keywords:** mHealth, diabetes mellitus, text message, mobile phone, SMS, self-management

## Abstract

**Background:**

The increasing prevalence of diabetes and costly long-term complications associated with poor glycemic control are issues facing health services worldwide. Diabetes self-management, with the support of health care providers, is critical for successful outcomes, however, frequent clinical contact is costly. Text messages via short message service (SMS) have the advantage of instant transmission at low cost and, given the ubiquity of mobile phones, may be the ideal platform for the delivery of diabetes self-management support. A tailored text message-based diabetes support intervention called Self-Management Support for Blood Glucose (SMS4BG) was developed. The intervention incorporates prompts around diabetes education, management, and lifestyle factors (healthy eating, exercise, and stress management), as well as blood glucose monitoring reminders, and is tailored to patient preferences and clinical characteristics.

**Objective:**

To determine the usability and acceptability of SMS4BG among adults with poorly controlled diabetes.

**Methods:**

Adults (aged 17 to 69 years) with type 1 (n=12) or type 2 diabetes (n=30), a hemoglobin A1c (HbA1c) over 70 mmol/mol (8.6%), and who owned a mobile phone (n=42) were recruited to take part in a 3-month pilot study of SMS4BG. At registration, participants selected the modules they would like to receive and, where appropriate, the frequency and timing of blood glucose monitoring reminders. Patient satisfaction and perceptions of the usability of the program were obtained via semistructured phone interviews conducted at completion of the pilot study. HbA1c was obtained from patient records at baseline and completion of the pilot study.

**Results:**

Participants received on average 109 messages during the 3-month program with 2 participants withdrawing early from the study. Follow-up interviews were completed with 93% of participants with all reporting SMS4BG to be useful and appropriate to their age and culture. Participants reported a range of perceived positive impacts of SMS4BG on their diabetes and health behaviors. HbA1c results indicated a positive impact of the program on glycemic control with a significant decrease in HbA1c from baseline to follow-up.

**Conclusions:**

A tailored text message-based intervention is both acceptable and useful in supporting self-management in people with poorly controlled diabetes. A randomized controlled trial of longer duration is needed to assess the efficacy and sustainability of SMS4BG.

##  Introduction

Globally, diabetes is a significant health issue with increasing incidence worldwide. There is a disproportionate burden of the disease among indigenous peoples internationally [1] and in New Zealand (NZ), where higher disease prevalence is seen in Māori compared with New Zealand Europeans [2]. With the growing incidence and prevalence, there is a subsequent growing burden of caring for those living with the disease. Substantial evidence indicates that good diabetes control provides significant benefit in relation to the reduction of risk of complications [3,4]. Given the costly and debilitating microvascular and macrovascular complications of poorly controlled diabetes, including renal failure, visual impairment, lower limb amputation, heart disease, and stroke, intensive and sustained individual effort is required to achieve optimum control. Given the large impact that individual behaviors have on diabetes control, such as diet, energy expenditure, blood glucose monitoring, medication adherence, and self-adjustment of insulin doses, the standard of diabetes care includes self-management education and support. For those with poor control, education and support needs to extend outside the clinic setting in order to sustain the behaviors needed to manage diabetes in the context of their daily lives. One way to extend self-management support beyond the clinic is through ecological momentary interventions (EMI), which are delivered during a person’s daily life providing “real-world” support in “real time” [5]. Mobile phones provide an ideal method for delivering EMI, as they are carried with most people most of the time, thereby maximizing their potential to optimize support for those in need.

Mobile health (mHealth) is the use of mobile devices, including mobile phones, to deliver health services and information [6]. The field of mHealth is growing with increasing support for its use in behavior change and disease management, including smoking cessation, weight loss, cardiac rehabilitation, and diabetes management [7]. Mobile phone ownership and use have continued to increase internationally and in New Zealand [8-10], with high penetration across all groups including hard-to-reach populations. All digital mobile phones provide short message service (SMS), also known as text messaging, with New Zealand having the highest use of SMS by head of population in 2011 compared to other Organisation for Economic Co-operation and Development (OECD) countries [11]. Given the high level of mobile phone ownership and the prolific use of SMS, this mode of communication appears an ideal platform for the delivery of health interventions.

Recent systematic reviews show that the majority of SMS-based behavior change interventions for disease management have positive short-term impacts on behavioral and clinical outcomes [7,12,13]. There is an increasing body of evidence supporting the use of mobile phones and SMS in the management of diabetes, including evidence for these interventions resulting in significant short-term improvements in glycemic control [14,15]. However, although studies to date have shown promising results, there are a lack of theoretically based comprehensive diabetes mHealth interventions [7,16]. Previous research has also highlighted the need to individually tailor messages [5,17-19], as well as to provide people with choices to increase their sense of control over the intervention [20]. To address these previous limitations, we developed and pilot-tested SMS4BG (Self-Management Support for Blood Glucose) a new tailored SMS self-management support program for adults with poorly controlled diabetes in New Zealand.

## Methods

### Study Design

#### Overview

A 3-month, nonrandomized pilot study was conducted between July and December 2013. All study documents and procedures were approved by the Health and Disability Ethics Committee (13/NTA/55).

#### Participants and Recruitment

Eligibility criteria included adults aged 16 to 70 years, a diagnosis of type 1 or type 2 diabetes mellitus, hemoglobin A1c (HbA1c) >70 mmol/mol (8.6%) within the last 12 months, mobile phone ownership, ability to provide informed consent, and ability to read English. An HbA1c result of greater than 70 mmol/mol (8.6%) was utilized as the definition of poorly controlled diabetes in this study, a level associated with increased risk for the development of diabetes complications. Recruitment was carried out across three primary health care practices, two secondary care hospitals, and one community-based organization in Auckland, New Zealand. Clinicians at each site identified potential participants and either enrolled them directly through the study website or referred them to a research assistant to complete registration. Registered participants then received an automated consent text message and were required to reply “Yes” to be enrolled in the program. The program was free to receive but if a participant replied, they were charged NZD $0.20 per message by their network provider. Participants were given a voucher (NZD $20.00) at the conclusion of the study to reimburse them for their time and any costs associated with replying to the messages.

#### Measures

At the end of the program all participants (including those that withdrew) were asked to complete questions about their satisfaction with the program, its usefulness and usability, and perceived positive impacts, via a semistructured telephone interview conducted by a research assistant. Engagement with the program was assessed using system-recorded responses to the blood glucose monitoring reminder messages. In addition, participants consented to the research team obtaining their HbA1c test results from their medical records to assess change in HbA1c from baseline to follow-up.

#### Statistical Analysis

Descriptive statistics were generated for baseline demographic and clinical characteristics, and measures of engagement with the system. Counts and percentages were reported for categorical variables, and means and standard deviations for continuous variables. To determine whether ratings of usefulness differed between ethnic groups and diabetes type, *t* tests were used. Change in HbA1c was calculated using the related-samples Wilcoxon signed-rank test.

### Intervention Development

SMS4BG was developed to provide self-management support for adults with poorly controlled diabetes. The content was developed by a multidisciplinary team, led by a health psychologist (RD) and public health physician (RW). The development followed the mHealth Development and Evaluation framework [21], which provides a process to guide the development and testing of mHealth interventions with a focus on implementation, use of behavioral change theory, and involvement of the target population.

The development of the content was informed by a review of the research literature, existing mHealth interventions (targeting diabetes management and related lifestyle behaviors), and current patient resources. The program was informed by two behavior change theories: Social Cognitive Theory [22] and the Common Sense Model [23]. Messages were designed to provide correct perceptions around diabetes and its management and to increase self-efficacy and perceived support for diabetes management. SMS4BG also utilized a number of different behavior change techniques (BCTs) [24] to support behavior change in relation to diabetes management: providing general information linking behavior to health, providing information on consequences, prompting intention formation, prompting barrier identification, providing general encouragement, prompting self-monitoring of behavior, providing feedback on performance, and stress management.

To accommodate personal preferences and clinical characteristics, SMS4BG was made up of modules including a core module that all participants received and additional optional modules. Clinician input determined optional module topics. The core module consisted of 2 messages per week on diabetes education, emotional encouragement, and illness perceptions (available in Māori and non-Māori versions). In addition to the core module, if registered as a smoker, the participant received an additional 1 message per month supporting smoking cessation. Participants could also opt to receive additional modules on topics relevant to diabetes management such as insulin, diet, exercise, stress management, and blood glucose monitoring reminders. A summary of the different SMS4BG modules can be seen in Table 1. There were a total of 180 different messages across all modules with the minimum number of messages a participant could receive being 30 messages over the 3-month period, unless they withdrew early. If the maximum number of modules and blood glucose monitoring reminders were selected, a participant could receive up to 461 messages over the 3-month period. All messages a participant received were unique with the exception of the blood glucose monitoring reminders for which there were 9 different reminder messages that they received.

The SMS4BG program was designed so that text messages were send-only (unidirectional) with the exception of the blood glucose monitoring reminders, which provided the option for participants to reply with their blood glucose test results. In addition to SMS, there was an accompanying website that patients and clinicians could log onto, allowing them to review a graphical display of the participant’s blood glucose responses sent into the system. The website also provided administrators with the ability to manage the message content and monitor message delivery. To enhance participant engagement, SMS4BG was personalized with the inclusion of each participant’s name in many of the messages. Individuals could also select the frequency and timing of blood glucose monitoring reminder messages—from 1 per week to up to 4 per day.

In New Zealand, there is a higher prevalence of diabetes in Māori in comparison with New Zealand Europeans [2]. To ensure the relevance of SMS4BG to this population, a Māori version of the core module was developed by the study’s Māori Advisory Group. The core messages were adapted to incorporate a greater focus on family (whānau), and incorporate key words in the Te Reo Māori language, although messages remained predominately in English.

Once developed, messages were reviewed by diabetes specialists and a selection of messages were pretested by people with diabetes. Feedback from this process was incorporated into the messages before they were finalized and entered into the system for testing. Following development, a pilot study was conducted which set out to assess the usability and acceptability of the text message support program in adults with poorly controlled diabetes.

**Table 1:** SMS4BG modules.

Module	Description	Participants	Example text message
Core	2 messages per week providing general motivation and support for diabetes management. Available in two versions: (1) Māori, and (2) non-Māori.	All	(1) “SMS4BG: Kia ora. Control of your glucose levels involves eating the right kai, exercise & taking your medication. Your whanau, doctor & nurse can help you.” (2) “SMS4BG: There is no quick fix to diabetes but with good management it will have less impact on your life and leave you more time to do the things you enjoy.”
Insulin	1 educational text message per week on insulin management for patients receiving insulin.	Available to participants prescribed insulin.	“SMS4BG: Unopened insulin should be kept in the fridge. Don’t use insulin that has changed color, lumpy, expired, cracked or leaking, has been frozen or too hot.”
Young adult	1 message per week around managing diabetes in the context of work/school and social situations.	Available to participants aged 16-24.	“SMS4BG: It’s important not to ignore a hypo. No one likes to be embarrassed, but ignoring a hypo can make you feel worse & can be more embarrassing.”
Smoking cessation	1 message per month encouraging participants to consider quitting smoking and providing details of services for support.	All participants who register as smokers.	“SMS4BG: Good management of your diabetes and your future health includes not smoking, call Quitline on 0800 778 778 for support.”
Lifestyle behavior	Up to 4 messages per week encouraging participants to set a lifestyle goal and supporting them to work toward this goal. Participants can receive one of these modules for 3 months. The three lifestyle modules are: (1) exercise, (2) healthy eating, and (3) stress and mood.	Available to all participants.	(1) “SMS4BG: Hi [name]. If you are finding it tough to keep up your exercise think about why good management of your diabetes is important to you.” (2) “SMS4BG: Healthy eating is an important part of your diabetes treatment and it will help you in controlling your blood glucose levels.” (3) “SMS4BG: Make sure you have fun activities scheduled regularly. Doing something enjoyable helps reduce stress & improves mood.”
Blood glucose monitoring	Reminders to test blood glucose, sent at a frequency selected by the participant (up to 4 per day), for which they are encouraged to reply by text message with their blood sugar readings.	Available to all participants required to monitor their blood glucose.	“SMS4BG: Hi [name]. Just a reminder it is time to check your blood glucose. Reply with the result.” If valid response received, “SMS4BG: Thank you for sending your result.”

## Results

A total of 44 potential participants were recruited, with 42 participants consenting to participate. Table 2 presents characteristics of the registered participants. Only 2 participants requested to end the program early, both during the third week of their messages. Of the 42 participants enrolled, 3 (7%) were lost to follow-up. Of these 3 participants, 2 could not be contacted, and the remaining participant’s phone had been disconnected.

**Table 2:** Participant characteristics (n=42).

Characteristic		n (%) or mean (SD)
Gender, n (%)		
	Male	20 (48)
**Ethnicity, n (%)**		
	NZ European	16 (38)
	Māori	15 (36)
	Pacific	3 (7)
	Other	8 (19)
Diabetes type, n (%)		
	Type 2	30 (71)
**Recruitment site, n (%)**		
	Primary care	22 (52)
	Secondary care	18 (43)
	Other	2 (5)
Age in years, mean (SD)		45.7 (13.1)
HbA1c in mmol/mol, mean (SD)		89 (22)

### Participant Engagement

Due to the choice of modules, participants received varying numbers of messages during the 3-month program. Table 3 presents a breakdown of the modules in which the participants were enrolled.

**Table 3:** Participants’ choices of SMS4BG modules (n=42).

Module		n (%)
**General**		
	Total	42 (100)
	Non-Māori	38 (90)
	Māori	4 (10)
Insulin		15 (36)
Young adult		3 (7)
Smoking cessation		10 (24)
**Lifestyle**		
	Total	34 (81)
	Exercise	12 (35)
	Healthy eating	12 (35)
	Stress	10 (30)
**Blood glucose monitoring reminder messages**		
	Total	34 (81)
	1/week	19 (56)
	3/week	4 (12)
	1/day	6 (18)
	2/day	3 (9)
	3/day	1 (3)
	4/day	1 (3)

Participants received on average 109 (range 8 to 437) text messages from the program during the 3-month period, with an average of 13 messages per week. This included on average 63 (range 8 to 93) send-only text messages per participant over the 3-month program. A total of 34 participants out of 42 (81%) opted to receive blood glucose monitoring reminders, receiving on average 58 (range 9 to 353) reminder messages each over the 3-month period. A total of 827 response messages were received from 26 (76%) of the 34 participants registered to receive reminders. Of those who responded to at least one reminder, participants on average responded to 57% of their reminder messages (range 1 to 99%). For those 8 participants that did not reply (8/34, 24%), cost was identified as the leading barrier. Only 4 (12%) of the 34 participants reported accessing their graph online to view their blood glucose results. The most frequently reported barriers were no access to computers or Internet and not responding to the messages, and as a result not having a graph to view.

### Patient Satisfaction and Usability

A summary of the results of the follow-up interviews is provided in Table 4. Participants reported high levels of satisfaction with SMS4BG—all (39/39, 100%) reported the program to be useful to some degree, and 97% (38/39) reported they would recommend the program to others with diabetes. When asked to rate how useful the messages were on a scale from 0 (*not at all useful*) to 5 (*extremely useful*), the mean rating was 3.94 (SD 0.98). Higher mean ratings of usefulness were seen in those with type 2 diabetes (4.21, SD 0.75) compared to those with type 1 diabetes (3.17, SD 1.17) (*P*=.004). Although not statistically significant, higher ratings of usefulness were found for Māori (4.13, SD 0.91) compared with New Zealand European (3.68, SD 0.99) (*P*=.23).

All participants were able to identify at least one positive impact of the program. The majority (32/39, 82%) of participants reported that the program had a positive impact on their overall blood glucose control. In addition, 49% (19/39) of all participants interviewed reported a positive impact of SMS4BG on their exercise habits, 59% (23/39) on their diet and eating behavior, and 67% (26/39) on their mood. Of the participants interviewed who received the exercise lifestyle module, 83% (10/12) reported a positive impact of SMS4BG on their exercise habits. Of those interviewed who received the healthy eating module, 82% (9/11) reported a positive impact on their diet and eating behavior. Of those interviewed who received the stress and mood module, 67% (6/9) reported a positive impact on their mood. Of those 10 who were registered as smokers, 3 (30%) participants reported that they had quit smoking during the program.

Suggestions for improvements in the program included making the program longer, allowing for two-way communication with health care professionals through the program, making it free to reply to the messages, allowing for greater choice in the timing of the messages, and greater personalization. Few technical issues were reported—of the 39 participants interviewed, 2 (5%) reported issues accessing their graph, and 8 (21%) participants reported that not having credit/money on the phone account meant they could not reply with their blood glucose test results.

**Table 4:** Results of the follow-up interviews (n=39).

Question		Response (“yes”), n (%)
Was SMS4BG useful?		39 (100)
Were the messages culturally appropriate?		39 (100)
Were the messages age appropriate?		39 (100)
**Do you think SMS4BG has had a positive impact on:**		
	Your overall BG control?	32 (82)
	Your frequency of BG monitoring?	30 (77)
	Your diet or eating?	23 (59)
	Your exercise?	19 (49)
	Your mood?	26 (67)
	Your perception of your diabetes?	19 (49)
	Your knowledge of diabetes?	16 (41)
Would you recommend SMS4BG to others with diabetes?		38 (97)

### Metabolic Control

Baseline HbA1c values were obtained for all participants, but follow-up results were only available for 26 (62%) of the 42 participants. A significant improvement in HbA1c was found from baseline (median 89.50 mmol/mol) to follow-up (median 71.00 mmol/mol, Wilcoxon signed-rank test *P*=.001) for the 26 participants out of 42 (62%) for whom complete data was available.

##  Discussion

This pilot study has established that SMS4BG is an acceptable and potentially useful tool for adults with poorly controlled diabetes. Perceived positive impacts of the program were complemented by a significant improvement in glycemic control at follow-up. This aligns with previous text message-based interventions in people with diabetes [14].

Further evidence of the acceptability of SMS4BG was seen in the follow-up interviews, with all participants reporting SMS4BG to be both culturally and age appropriate. Participants ranged in age from 17 to 69 years and over half of the participants were of Māori (15/42, 36%), Pacific (3/42, 7%) or Asian decent (4/42, 10%). This indicates that this type of technology is not limited by demographic characteristics and the text message content was relevant to a wide range of people with poorly controlled type 1 and type 2 diabetes.

Most participants were satisfied with the number and frequency of the messages they received, which may be due to participants being involved in the selection of the modules they received and, therefore, having some degree of control over the number of messages they received.

Although visual feedback was provided in the form of a graph of submitted blood glucose results, this feature was not utilized by the majority of participants. The leading barrier for not accessing the graph was lack of Internet access either at home or on their mobile phones. Previous studies have reported greater improvements in HbA1c with combined mobile and Internet-based interventions compared to studies utilizing mobile intervention alone [14]. Our findings are in contrast to this and highlight that lack of Internet access can reduce participant access to features of the interventions. Other methods for providing feedback should be investigated, such as sending graphs via multimedia messaging service (MMS). Although it was free to take part in the pilot study, the barrier of cost of replying to text messages (NZD $0.20) was identified as preventing a number of participants from responding with their blood glucose results and, therefore, feedback was not available to them. To ensure that SMS4BG is able to be fully utilized by all, removing the cost of reply messaging may be needed if rolled out within a health care setting.

Strengths of the SMS4BG program included that it was theoretically informed, system initiated (ongoing intervention not dependent on participant behaviors), personally tailored, and provided participant choice. Many previous diabetes text messaging programs have had limited reach or were designed specifically for one diabetes type, age group, or single diabetes management behavior. SMS4BG was designed for adults of all ages with both poorly controlled type 1 and type 2 diabetes and provided support for self-management and encouragement in people’s everyday lives rather than focusing on specific diabetes-related tasks. In addition, SMS4BG utilized simple technology and, therefore, had less potential for technical issues that have been a limitation in previous mHealth studies.

Another strength of the current study was the inclusion of an indigenous version. With a higher prevalence of diabetes seen in Māori compared to NZ Europeans [2], diabetes interventions need to be both relevant and culturally appropriate to this group. There were two programs that Māori could choose from and although only 4 participants chose to register for the Māori version, no Māori participants withdrew from the program. This acknowledgement of identity may have assisted with retention of the Māori participants. In addition, the inclusion of motivational messages linking diabetes management to family (whānau) aligns with the importance of whānau to the well-being of Māori [25]. Although not significant, the higher ratings of usefulness of SMS4BG by Māori participants compared with New Zealand Europeans warrants further investigation. In addition, future development of the program could incorporate other cultural versions, including one for Pacific peoples.

This study had several limitations, including the absence of a control group and a small sample size. Although positive change in glycemic control was seen without a control group or adequate sample size, this difference must be interpreted with caution. The lack of complete follow-up HbA1c results limits the generalizability of the improved glycemic control results. The target population (poorly controlled) were likely not attending medical appointments as regularly as guidelines state and, therefore, the lack of clinical results could be expected. Future studies could include text messages around the importance of HbA1c tests and reminders to go for tests as a way of potentially overcoming this issue. The pilot study was of short duration and as diabetes is a condition requiring long-term management, longer interventions may be more appropriate. Another limitation was the lack of follow-up to assess whether any effects of SMS4BG were maintained beyond the program itself. A larger and longer-term randomized controlled trial will need to be carried out to establish the efficacy of SMS4BG on self-management behaviors, self-efficacy and clinical outcomes, and its sustainability and cost-effectiveness.

The current study adds to the evidence for the use of mHealth in delivering personally tailored diabetes self-management support and, particularly, the use of text messaging as a medium of delivery. The positive pilot study results indicate that this type of broad reaching EMI could be successful in engaging adults with poorly controlled type 1 or type 2 diabetes and assisting with improved diabetes self-management. Further refinement of SMS4BG is needed based on the pilot study feedback, followed by a larger randomized control trial to determine its efficacy.

## Abbreviations

BCT: behavior change technique
EMI: ecological momentary interventions
HbA1c: hemoglobin A1c
mHealth: mobile health
MMS: multimedia messaging service
NZ: New Zealand
OECD: Organisation for Economic Co-operation and Development
SMS: short message service
SMS4BG: Self-Management Support for Blood Glucose
